# Association between Dapagliflozin, Cardiac Biomarkers and Cardiac Remodeling in Patients with Diabetes Mellitus and Heart Failure

**DOI:** 10.3390/life13081778

**Published:** 2023-08-20

**Authors:** Andrew Xanthopoulos, Nikolaos Katsiadas, Spyridon Skoularigkis, Dimitrios E. Magouliotis, Niki Skopeliti, Sotirios Patsilinakos, Alexandros Briasoulis, Filippos Triposkiadis, John Skoularigis

**Affiliations:** 1 Department of Cardiology, University Hospital of Larissa, 41110 Larissa, Greece; spiros6231998@gmail.com (S.S.); nskopeliti@gmail.com (N.S.); ftriposkiadis@gmail.com (F.T.); 2 Department of Cardiology, Konstantopoulio General Hospital, 14233 Nea Ionia, Greece; 3 Unit of Quality Improvement, Department of Cardiothoracic Surgery, University of Thessaly, 41110 Larissa, Greece; dmagouliotis@gmail.com; 4 Department of Therapeutics, Heart Failure and Cardio-Oncology Clinic, National and Kapodistrian University of Athens, 11527 Athens, Greece; alexbriasoulis@gmail.com

**Keywords:** sodium glucose cotransporter-2 inhibitors, dapagliflozin, remodeling, diabetes mellitus, heart failure

## Abstract

Sodium–glucose cotransporter-2 inhibitors (SGLT2is) are a relatively new class of antidiabetic drugs that have shown favorable effects in heart failure (HF) patients, irrespective of the left ventricular ejection fraction (LVEF). Recent studies have demonstrated the beneficial effects of empagliflozin on cardiac function and structure; however, less is known about dapagliflozin. The purpose of the current work was to investigate the association between the use of dapagliflozin and cardiac biomarkers as well as the cardiac structure in a cohort of patients with HF and diabetes mellitus (DM). The present work was an observational study that included 118 patients (dapagliflozin group n = 60; control group n = 58) with HF and DM. The inclusion criteria included: age > 18 years, a history of DM and HF, regardless of LVEF, and hospitalization for HF exacerbation within the previous 6 months. The exclusion criteria were previous treatment with SGLT2i or glucagon-like peptide-1 receptor agonists, a GFR< 30 and life expectancy < 1 year. The evaluation of patients (at baseline, 6 and 12 months) included a clinical assessment, laboratory blood tests and echocardiography. The Mann–Whitney test was used for the comparison of continuous variables between the two groups, while Friedman’s analysis of variance for repeated measures was used for the comparison of continuous variables. Troponin (*p* < 0.001) and brain natriuretic peptide (BNP) (*p* < 0.001) decreased significantly throughout the follow-up period in the dapagliflozin group, but not in the control group (*p* > 0.05 for both). The LV end-diastolic volume index (*p* < 0.001 for both groups) and LV end-systolic volume index (*p* < 0.001 for both groups) decreased significantly in the dapagliflozin and the control group, respectively. The LVEF increased significantly (*p* < 0.001) only in the dapagliflozin group, whereas the global longitudinal strain (GLS) improved in the dapagliflozin group (*p* < 0.001) and was impaired in the control group (*p* = 0.021). The left atrial volume index decreased in the dapagliflozin group (*p* < 0.001) but remained unchanged in the control group (*p* = 0.114). Lastly, the left ventricular mass index increased significantly both in the dapagliflozin (*p* = 0.003) and control group (*p* = 0.001). Dapagliflozin, an SGLT2i, was associated with a reduction in cardiac biomarkers and with reverse cardiac remodeling in patients with HF and DM.

## 1. Introduction

There is a bidirectional relationship between heart failure (HF) and diabetes mellitus (DM), as DM is a significant risk factor for developing a new HF episode and vice versa [1,2,3]. DM affects the heart through various mechanisms that result in significant structural and functional changes in the myocardium [3,4,5]. In patients with HF, the concomitant presence of DM has been associated with a worse prognosis [5,6].

Large, randomized trials and meta-analyses have revealed an association between sodium–glucose cotransporter-2 inhibitors (SGLT2is) and a reduction in the risk of death and rehospitalization in patients with HF with or without the presence of DM [6,7]. SGLT2is are a relatively new class of antidiabetic drugs, whose main benefit arises from their pleiotropic effects on the heart [6,7]. It has been suggested that one of the mechanisms through which the cardioprotective effect of SGLT2is is achieved is the reversal of cardiac remodeling [8]. Recent studies have demonstrated the beneficial effects of empagliflozin on cardiac function and structure [9,10,11,12]. However, despite the promising results of the randomized IDDIA (The Impact of Dapagliflozin on Left Ventricular Diastolic Dysfunction in Patients with Type 2 Diabetes Mellitus) trial, which showed that treatment with SGLT2i dapagliflozin improved left ventricular (LV) diastolic dysfunction, in 60 patients with type 2 DM (T2DM) [13], existing studies have reported conflicting results regarding the association of dapagliflozin and cardiac remodeling or function in patients with HF and T2DM [14,15]. In particular, in the REFORM study (Safety and Effectiveness of SGLT-2 Inhibitors in Patients With Heart Failure and Diabetes), a randomized, double-blind, single-center study, in 56 patients with DM and HF with LVEF < 45%, dapagliflozin administration for 12 months had no effect on LV remodeling (i.e., left atrial volume index/LAVI, left ventricular end-systolic volume index/LVESVI, left ventricular end-diastolic volume index/LVEDVI and left ventricular mass index/LVMI) [14]. On the contrary, a prospective multicenter study of 58 T2DM patients with stable HF in Japan reported an association between the use of dapagliflozin and a decrease in LAVI and LVMI (both *p*  <  0.001), but no association with brain natriuretic peptide (BNP) 6 months after the administration of dapagliflozin [15]. The recent DAPA-MODA trial (NCT04707352) was a multicenter, single-arm, open-label, prospective study that demonstrated the association between dapagliflozin use and the reduction in cardiac volumes, as well as cardiac biomarkers (i.e., NT-proBNP), in a cohort of 162 stable chronic HF patients (21.6% diabetics). However, the study observations were limited by the lack of a control group [16].

Taken all together, there is conflicting evidence regarding the association between dapagliflozin, cardiac biomarkers and cardiac remodeling in patients with HF and type 2 DM. Therefore, the aim of the present study was to examine these associations utilizing “real-world” data.

## 2. Materials and Methods

### 2.1. Study Population

**Figure 1:**
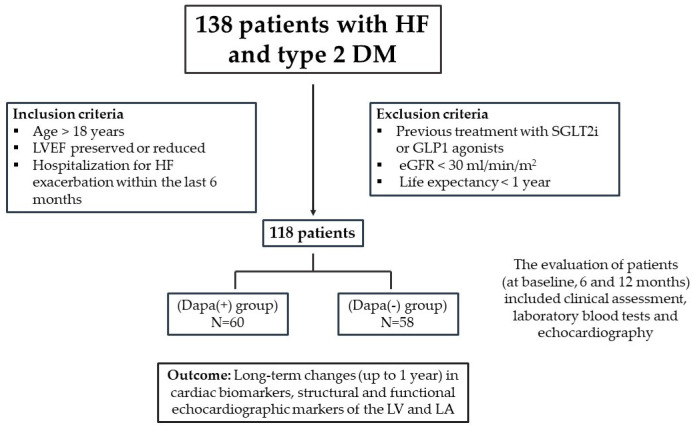
Study flowchart.

The present work was a single-center observational study of 138 consecutive patients with HF and T2DM who were examined in the ambulatory clinic from 15 June 2020 to 15 June 2021 (Figure 1, study flowchart). All patients had to be over 18 years of age with a known history of T2DM and HF, regardless of left ventricular (LV) ejection fraction (LVEF), and hospitalization for HF exacerbation within the last 6 months. Exclusion criteria were previous treatment with SGLT2i or glucagon-like peptide 1 (GLP1) agonists, an estimated glomerular filtration rate (GFR) < 30 mL/min/m^2^ and life expectancy < 1 year. After the implementation of inclusion and exclusion criteria, the 118 patients that remained were divided into two cohorts. The first cohort consisted of 60 patients with HF and DM type 2 (T2DM) who started dapagliflozin (10 mg once daily on top of antidiabetic treatment, Dapa (+) group) at baseline and the second cohort consisted of 58 patients with HF and T2DM who continued antidiabetic treatment without dapagliflozin or another SGLT2i (the Dapa (−) group). The follow-up period was 12 months. The treatment was based on the physician’s discretion.

The study was conducted in accordance with the Declaration of Helsinki and approved by the institutional review board (or ethics committee) of the University of Thessaly (protocol code: 2001; date of approval: 9 April 2020). Informed consent was obtained from all subjects involved in the study.

### 2.2. Patient Assessment

The evaluation of patients (at baseline, 6 and 12 months) included a clinical assessment, laboratory blood tests and echocardiography. The echocardiography was reviewed by two independent echocardiographers with the use of a GE, Healthcare Vivid E95 device. Standard echocardiographic measurements were obtained in accordance with the current guidelines of the European Association of Cardiovascular Imaging [17]. The LV and left atrial (LA) dimensions were obtained through linear measurements from 2D echoes. The LVEF, LV and LA volumes were measured using the biplane method of discs (modified Simpson’s rule) with 2D echoes. The LV mass was estimated by using the formula proposed by Devereux et al. [18]. The LV end-diastolic volume index (LVEDVi), LV end-systolic volume index (LVESVi), LA volume index (LAVi) and LV mass index were calculated by dividing the LVEDV, LVESV, LA volume and LV mass by the body surface area (BSA). A speckle-tracking strain analysis was performed for each patient to evaluate the LV longitudinal function, which was assessed in terms of the global longitudinal strain (GLS). The longitudinal speckle-tracking strain was calculated by applying an automated contouring detection algorithm, and the manual adjustments of regions of interest were performed where necessary. Brain natriuretic peptide (BNP) and troponin were measured with a Dimension EXL Siemens analyzer.

### 2.3. Outcomes

The aim of the present analysis was to examine and compare the long-term changes (up to 1 year) in cardiac biomarkers, as well as structural and functional echocardiographic markers of the LV and LA between the group of patients who received dapagliflozin and the control group.

### 2.4. Statistical Analysis

Quantitative variables were expressed as the median (25th percentile to 75th percentile), whereas categorical variables as percentages. The Mann–Whitney test was used for the comparison of continuous variables between the two groups, while Friedman’s analysis of variance for repeated measures was used for the comparison of continuous variables. Proportions were compared using Fisher’s exact test. The influence of differences in baseline characteristics on the results was examined with a stepwise linear regression analysis with the change in the LVEF during follow-up as the dependent variable. All reported *p* values were two-tailed. Statistical significance was set at *p* < 0.05 and analyses were conducted using SPSS statistical software (IBM Corp. Released 2022. IBM SPSS Statistics for Windows, Version 29.0. Armonk, NY, USA: IBM Corp).

## 3. Results

### 3.1. Patient Characteristics

The baseline characteristics of the study population are presented in Table 1. A total of 118 patients (21(17.8%) females) were included with a median age of 69.5 years. Interestingly, 60 (50.8%) received dapagliflozin. Patients in the Dapa (+) group were younger compared with the control group. On the other hand, patients in the Dapa (−) group had lower HbA1C and higher urea values. The other baseline characteristics (i.e., clinical characteristics, laboratory parameters and risk factors/comorbidities) including medical treatment were not significantly different between the two groups.

### 3.2. Hemodynamic Changes and Biomarkers by Time Point and by Group

The patients’ hemodynamic changes and biomarkers by time point and by group are presented in Table 2. The heart rate and systolic blood pressure decreased significantly in both groups. However, the diastolic blood pressure remained unchanged. Troponin and BNP decreased in the Dapa (+) group, contrary to the Dapa (−) group, in which it remained unchanged.

### 3.3. Echocardiographic Changes by Time Point and by Group

**Figure 2:**
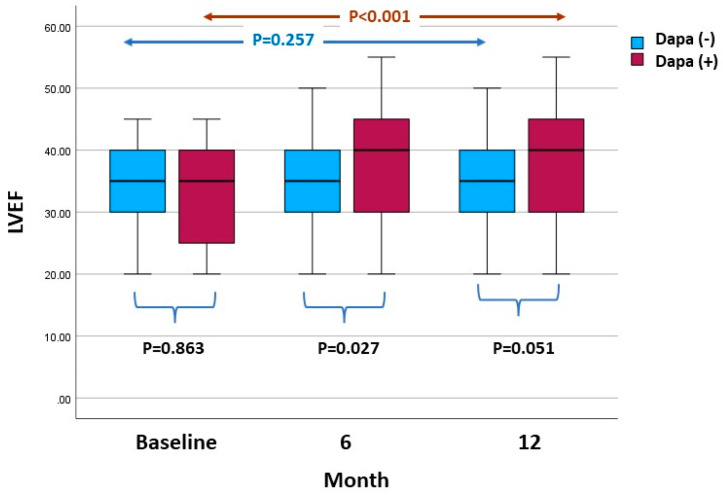
Left ventricular ejection fraction (LVEF) values at various time points (baseline, 6 months and 12 months) in the dapagliflozin (Dapa (+)) group and the non-dapagliflozin (Dapa (−)) group.

**Figure 3:**
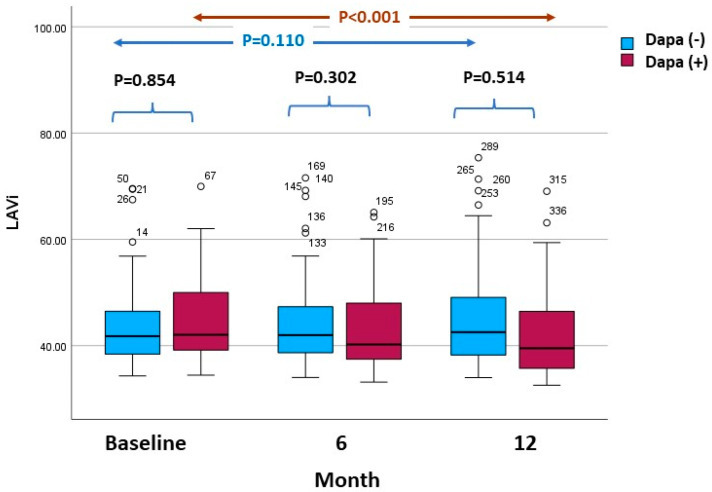
Left atrial volume index (LAVi) values at various time points (baseline, 6 months and 12 months) in the dapagliflozin (Dapa (+)) group and the non-dapagliflozin (Dapa (−)) group.

LVEDVis and LVESVis lowered during the follow-up in both groups (Table 3). The LVEF did not change significantly over the follow-up period in the Dapa (−) group, while it increased significantly over the follow-up period in the Dapa (+) group (Figure 2). The GLS increased significantly throughout the follow-up period in the Dapa (−) group, while it decreased significantly in the Dapa (+) group. The LAVi decreased significantly in the Dapa (+) group, whereas it remained unchanged in the control group (Figure 3). The LV mass index increased significantly throughout the follow-up period in both groups.

Table 4 shows that the effect on the left ventricular function of age, HbA1C and urea, which differed between the two study groups at baseline, was negligible compared with the effect of the intervention.

## 4. Discussion

In the present analysis, the use of SGLT2i dapagliflozin for 12 months in patients with HF and DM was associated with a decrease in troponin and BNP compared to the control group, as well as a reduction in left ventricular (LVESVi and LVEDVi) and left atrial (LAVi) volumes. The dapagliflozin-treated patients were also found to have an improvement in LV function indices (LVEF and GLS).

DM is one of the main risk factors for HF development, as it affects the heart through various pathophysiological pathways [1,4]. HF in DM is a heterogeneous syndrome depending on diverse factors, in which disease progression is associated with a dynamic evolution of functional and structural changes, creating a spectrum of phenotypes with overlapping and distinct characteristics [1]. For example, through the macroangiopathy it causes, it increases the incidence of CAD and, by extension, the risk of developing ischemic cardiomyopathy and HF [2]. Hypertension, a common risk factor of HF development, is a frequently coexisting morbidity in patients with DM [1]. Various common pathophysiological mechanisms contribute to the coexistence of HTN and DM, including, but not limited to, hyperinsulinemia, abnormal renal sodium handling, the overactivation of the renin–angiotensin–aldosterone system, inflammation, oxidative stress and endothelial cell dysfunction [1]. DM is also associated with immediate structural changes in the myocardium, even in the absence of CAD, as it increases interstitial fibrosis and causes myocardial hypertrophy, resulting in the development of diastolic dysfunction, increased intraventricular pressures and HFpEF [3]. At a more advanced stage, and also due to microvascular disorders, a vicious cycle is created between functional disorders of the LV, an increase in its volumes and, finally, the development of HFrEF [4]. Cardiac remodeling is defined as changes in cardiac geometry and/or function over time, which can be measured through changes of cardiac chamber dimensions, volumes, mass and functions at serial imaging examinations, but mostly with echocardiography [19]. Together with imaging findings, circulating biomarkers (most notably BNP and troponin levels) may be helpful in this respect [19]. The underlying mechanisms of cardiac remodeling are complex, involving molecular events within cells and the interstitium, together acting to alter the shape, size and mass of the heart after cardiac injury [8]. Cardiac remodeling in patients with DM and HF is one of the main issues associated with increased mortality and morbidity in these patients, and is a therapeutic target [20]. SGLT2is block sodium/glucose cotransporter-2 located in the early proximal renal tubule, which leads to an increased urinary glucose excretion and, subsequently, decreased serum glucose concentrations [21]. SGLT2is have been demonstrated to reduce major adverse cardiovascular events and hospitalization for HF [21]. These cardioprotective benefits of SGLT2i have been suggested to be mediated through their pleiotropic actions that appear to affect cardiac function [22].

Both BNP and troponin are important biomarkers for patients with HF, as their elevated values have been associated with an increased risk of rehospitalization and death [23,24]. Few data exist regarding the effect of dapagliflozin on cardiac biomarkers. In the study by Soga et al., dapagliflozin was shown to decrease BNP levels only in patients with baseline levels above 100 [15]. The authors speculated that SGLT2is may have the potential to result in LV unloading in the case of HF patients with an LV load at a certain level. Berg et al., in an analysis of DAPA-HF (*Study to Evaluate the Effect of Dapagliflozin on the Incidence of Worsening Heart Failure or Cardiovascular Death in Patients With Chronic Heart Failure*) found a nonsignificant tendency to attenuate the increase in hs-troponin I in patients who received dapagliflozin, reflecting a possible reduction in myocardial injury over time [25]. In the present study, the use of dapagliflozin was associated with a decrease in BNP and troponin levels in the dapagliflozin group, but not in the control group.

It is very important to echocardiographically identify structural abnormalities in patients with HF and DM, as these have been correlated with prognosis in these patients [20]. Although it seems that the use of SGLT2i and, more specifically, empagliflozin, in patients with HF and DM is associated with the reduction in LV volumes and mainly of LVESV and LVESVi, few data exist regarding the effect of dapagliflozin on volumes (LVEDV, LVEDVi, LVESV and LVESVi) and the size (LVEDD) of the LV [10,12]. Xue et al. showed that patients with ST elevation myocardial infarction (STEMI) and DM treated with dapagliflozin in addition to standard therapy exhibited a reduction in LVEDV and LVESV (*p* < 0.05) compared to the control group after 24 weeks of treatment [26]. In the present study, we found that the use of dapagliflozin in patients with DM and HF was associated with a reduction in the volumes (LVESVi and LVEDVi) of the LV during the 1-year follow-up. Observational studies have associated dapagliflozin with an improvement in diastolic function in patients with HF and, thus, a reduction in the LA volume [27,28]. A prospective multicenter study, including 58 patients with DM and stable HF, showed that dapagliflozin administration for 6 months improved the LV diastolic parameters and decreased LAVi [31 to 26 mL/m^2^ (*p* = 0.001)] [15]. In the present study, the use of dapagliflozin was associated with a significant reduction in the LAVi compared to the control group at follow-up.

A common structural disorder often seen in patients with DM and HF is LV hypertrophy [29]. Recent data support that SGLT2i use reverses LV hypertrophy, although the underlying mechanism of action remains poorly understood [30]. In the DAPA-LVH trial (Does Dapagliflozin Regress Left Ventricular Hypertrophy In Patients With Type 2 Diabetes?), a randomized study of 66 patients with type 2 DM, dapagliflozin administration for 12 months was associated with a reduction in the LV mass of −2.82 g (95% confidence interval (CI): −5.13 to −0, 51, *p* = 0.01) attributed to the beneficial effects of dapagliflozin on the hemodynamic and metabolic factors [31]. However, no reduction was observed when the researchers examined the LV mass index values [31]. The aforementioned results contradicted the study by Soga et al., which showed that the use of dapagliflozin was associated with a decrease in the LV mass index (75.0 to 67.0 g/m^2^ (*p* < 0.001)) [15]. In the present study, the LV mass index values increased in both groups.

Both impaired LVEF and GLS have been associated with a negative prognostic outcome in patients with HF and DM, and are typically late echocardiographic findings in these patients [32,33,34]. Regarding the effect of dapagliflozin on the LVEF, there have been conflicting results. In the ADD DAPA study (ADDition of DAPAgliflozin, Sodium–Glucose Cotransporter-2 Inhibitor to Angiotensin Receptor Blocker-Neprilysin Inhibitors Non-Responders in Patient with Refractory Heart Failure with Reduced Ejection Fraction), a retrospective analysis of 104 patients with HFrEF, dapagliflozin administration for 6 months was found to improve the LVEF [(29 ± 4% to 38 ± 5%; +9.00 ± 0.628%; *p* < 0.001)] [35]. In contrast, in the REFORM study, dapagliflozin administration for 12 months had no effect on the LVEF [14]. In the present study, patients receiving dapagliflozin showed an improvement in the LVEF and GLS compared to the control group at both 6 and 12 months. This observation denoted that dapagliflozin administration had beneficial effects on markers of systolic cardiac function up to 12 months of follow-up [36,37,38]. Tanaka et al. showed that in a sample of 53 patients with DM and HF, there was an improvement in the GLS (15.5 ± 3.5% to 16.9 ± 4.1% (*p* < 0.01)) after receiving 6 months of dapagliflozin [39].

Based on the above results, we concluded that dapagliflozin is significantly associated with the reversal of LV remodeling. The reversal of cardiac remodeling is associated with clinical improvement, a better quality of life as well as a reduction in the risk of hospitalizations and death from HF [19,40,41]. Cardiac remodeling is a complex process involving various pathophysiological pathways, including inflammation, oxidative stress, metabolic abnormalities, mitochondrial dysfunction, autophagy and apoptosis, resulting in myocyte loss, cardiac hypertrophy and interstitial fibrosis [42,43,44]. Several studies have demonstrated that the use of SGLT2is, especially empagliflozin, was associated with an improvement in markers of cardiac function, confirming the importance of SGLT2 inhibition towards the reversal of cardiac remodeling [26]. The mechanisms involved in the reversal of cardiac remodeling using SGLT2i have not been fully established yet, but several hypotheses exist [45,46]. The presence of the SGLT2 receptor in the human cardiomyocyte is still debatable [47]. However, previous experimental and clinical studies have suggested several potential mechanisms that could be classified into four main categories [48]. Firstly, SGLT2is exhibit favorable hemodynamic and vascular effects that are mediated by a number of mechanisms, including osmotic diuresis, natriuresis, plasma and interstitial fluid volume reduction, reduction in arterial stiffness and sympathetic nervous system activity, as well as the improvement in endothelial function [46,48,49]. These effects have been shown to reduce cardiac preload and afterload, thus, could mitigate the LV stretch and wall stress, leading to a reduction in the LV volume. Secondly, SGLT2is were shown to have direct favorable renal effects. These favorable effects are mediated by mechanisms such as the reduction in the renin–angiotensin system activation, intraglomerular pressure, renal oxidative stress and the increase in erythropoietin levels, which, in turn, leads to the increase in hematocrit levels, a very important cardioprotective mechanism [50,51]. Thirdly, and most importantly, they perform direct favorable cardiac effects via several pathophysiological pathways. SGLT2 inhibition can alleviate cardiac inflammation by modulating the phenotype of macrophages and can reduce cardiac inflammation via inhibiting the activation of the nucleotide-binding domain-like receptor protein 3 (NLRP3) inflammasome [8]. Furthermore, they can improve myocardial energetics via the modulation of nutrient availability in myocardial cells and can decrease epicardial fat accumulation [48,50,51]. More specifically, in experimental models, SGLT2is prevented a decrease in cardiac function and increased cardiac ATP production without changing the overall metabolic efficiency [48]. This increase in cardiac energy production was the result of increased glucose oxidation and lower free fatty acid oxidation without changes in ketone body oxidation [48]. Moreover, another proposed mechanism for the beneficial effect of SGLT2is is the inhibition of the sodium–hydrogen exchanger (NHE1) activity, which is up-regulated both in DM and HF [48,50,51]. By inhibiting the NHE1 receptors, SGLT2is may protect the heart from toxic intracellular Ca^2^+ overload [22,52,53]. The DAPA-MEMRI trial (DAPA-MEMRI) could assess the role of altered calcium handling in diabetic cardiomyopathy and HF and determine the effects of SGLT2i therapy on cardiac calcium homeostasis (NCT04591639). The above mechanisms could lead to a decrease in myocardial hypertrophy and fibrosis and, therefore, play an important role in reversing cardiac remodeling [48,50,51]. Fourthly, the last proposed category of SGLT2is’ beneficial effects is the improvement of metabolic factors [50,51]. SGLT2is decrease insulin resistance, hyperinsulinemia and increase glucose excretion, as well as circulating ketone body levels; this may provide an additional source of energy to sustain the cardiac contractile function [49,50,51]. Randomized controlled studies are certainly needed to better characterize the above mechanisms.

## 5. Study Limitations

The sample size was not large (118 patients), even if each patient completing the follow-up had three measurements (at baseline, 6 and 12 months). Another limitation was the lack of a double-blind and placebo group design, as the present was a single-center observational study, although patients received optimized medical therapy. Females were underrepresented in both study groups. Therefore, probable sex-dependent alterations in adverse cardiac remodeling were not considered in the current work. However, sex was unrelated to the LVEF both in the Dapa (+) and in the Dapa (−) groups (R = 0.168, *p* = 0.207 and R = 0.195, *p* = 0.162, respectively). Consequently, the results of this study should be evaluated with caution and used as a basis for larger studies.

## 6. Conclusions

Dapagliflozin, an SGLT2i, was associated with an improvement in the LV and LA structural and functional echocardiographic markers in patients with HF and DM. In addition, dapagliflozin was associated with a reduction in biomarkers, such as troponin and natriuretic peptides. The abovementioned findings may explain the beneficial effects of dapagliflozin in patients with HF and T2DM. Large randomized mechanistic trials examining the role of SGLT2is in those patients are urgently needed.

## Figures and Tables

**Table 1:** Baseline characteristics.

Variable	Dapa (+) (n = 60)	Dapa (−) (n = 58)	*p* Value
Clinical characteristics			
Age (years)	67.00 (62.25–73.00)	73.50 (66.50–78.25)	0.006
Sex (females, %)	9 (15%)	12 (20.7%)	0.476
Body mass index (kg/m^2^)	27.69 (25.34–31.65)	27.76 (26.17–30.09)	0.715
Heart rate (bpm)	69.50 (65.00–75.00)	74.00 (66.50–82.25)	0.099
Systolic blood pressure (mmHg)	125.50 (113.25–139.75)	128.00 (111.50–142.75)	0.998
Diastolic blood pressure (mmHg)	75.50 (69.25–85.00)	75.00 (67.25–84.00)	0.705
Laboratory work up			
Hemoglobin (g/dL)	13.15 (11.40–14.67)	13.2 (11.30–14.40)	0.854
Hematocrit (%)	41.15 (35.82–45.40)	39.75 (38.80–43.20)	0.854
Red blood cell distribution width (%)	14.60 (13.52–17.10)	14.50 (13.60–16.82)	0.854
Glucose (mg/dL)	151.50 (127.00–194.50)	156.00 (129.50–178.00)	0.581
HBA1c (%)	7.45 (7.10–8.30)	6.80 (6.37–7.62)	0.009
Urea (mg/dL)	45.00 (37.25–57.75)	55.00 (40.00–78.50)	0.010
Creatinine (mg/dL)	1.01 (0.80–1.24)	1.16 (0.91–1.50)	0.099
eGFR (mL/min/1.73 m^2^)	73.48 (56.83–97.27)	61.10 (47.77–77.69)	0.197
Sodium (mmol/L)	140.00 (137.25–142.00)	139.00 (138.00–142.00)	0.740
Potassium (mmol/L)	4.20 (4.00–4.57)	4.15 (3.77–4.50)	0.977
Troponin (ng/mL)	0.04 (0.04–0.05)	0.04 (0.04–0.04)	0.453
BNP (pg/mL)	166.20 (98.50–342.25)	207.00 (104.25–494.75)	0.581
Risk factors/comorbidities *			
Coronary artery disease (%)	55	56	0.261
Hypertension (%)	52	54	0.247
Dyslipidemia (%)	56	56	0.426
Atrial fibrillation (%)	15	20	0.260
Valvular heart disease (%)	5	6	0.707
Peripheral arterial disease (%)	14	14	0.918
Stroke (%)	7	10	0.389
Smoking (%)	23	16	0.215
Medications			
Beta-blockers (%)	56	54	0.960
ACEi/ARBs (%)	45	44	0.913
Sacubitril/Valsartan (%)	13	11	0.716
Mineralocorticoid receptor antagonists (%)	41	37	0.602
Furosemide (%)	40	39	0.947
Dipeptidyl Peptidase IV (DPP IV) Inhibitors (%)	24	30	0.201
Metformin	54	50	0.524
Thiazolidinediones (%)	1	3	0.293
Sulfonylureas (%)	3	5	0.434
Insulin (%)	21	25	0.367

* Risk factors/comorbidities were obtained based on the patients’ medical histories.

**Table 2:** Hemodynamic changes and biomarkers.

Variable	Baseline	6 Months	12 Months	*p* Value	95% Confidence Interval of *p* Value
Heart rate (beats per minute)					
Dapa (+)	69.50 (65.00–75.00)	68.00 (65.00–72.00)	64.50 (62.00–69.00)	<0.001	0.000–0.000
Dapa (−)	74.00 (66.50–82.25)	74.00 (69.00–78.50)	71.00 (64.00–77.50)	0.024	0.020–0.028
Systolic blood pressure (mm Hg)					
Dapa (+)	125.50 (113.25–139.75)	125.00 (112.00–132.00)	123.00 (111.75–130.00)	<0.001	0.000–0.000
Dapa (−)	128.00 (111.50–142.75)	129.00 (112.00–135.00)	125.00 (110.00–132.50)	0.010	0.007–0.012
Diastolic blood pressure (mm Hg)					
Dapa (+)	75.50 (69.25–85.00)	74.00 (68.75–82.00)	71.00 (68.00–76.00)	0.056	0.050–0.062
Dapa (−)	75.00 (67.25–84.00)	74.00 (69.00–84.5)	71.00 (64.00–79.50)	0.131	0.122–0.139
Troponin (ng/mL)					
Dapa (+)	0.04 (0.04–0.05)	0.04 (0.04–0.04)	0.04 (0.04–0.04)	<0.001	0.000–0.000
Dapa (−)	0.04 (0.04–0.04)	0.04 (0.04–0.04)	0.04 (0.04–0.04)	0.119	0.110–0.127
Brain Natriuretic Peptide (pg/mL)					
Dapa (+)	166.20 (98.50–342.25)	108.00 (67.25–218.00)	92.50 (55.50–152.25)	<0.001	0.000–0.000
Dapa (−)	207.00 (104.25–494.75)	152.00 (75.35–478.00)	156.00 (69.50–607.50)	0.143	0.134–0.152

**Table 3:** Echocardiographic changes.

Variable	Baseline	6 Months	12 Months	*p* Value	95% Confidence Interval of *p* Value
Left ventricular end-diastolic volume index (mL/m^2^)					
Dapa (+)	56.17 (49.65–63.17)	55.27 (49.88–63.02)	53.78 (49.58–61.83)	<0.001	0.000–0.000
Dapa (−)	55.91 (46.09–61.19)	56.34 (46.98–68.28)	58.29 (47.5–69.70)	<0.001	0.000–0.000
Left ventricular end-systolic volume index (mL/m^2^)					
Dapa (+)	35.71 (29.78–41.28)	33.51 (28.47–40.09)	32.56 (27.79–39.59)	<0.001	0.000–0.000
Dapa (−)	36.80 (27.70–43.26)	35.54 (28.73–47.04)	34.74 (28.41–48.25)	<0.001	0.000–0.000
Left ventricular ejection fraction (%)					
Dapa (+)	35 (25–40)	40 (30–45)	40 (30–45)	0.001	0.000–0.000
Dapa (−)	35 (30–40)	35 (30–40)	35 (30–40)	0.279	0.267–0.290
Global longitudinal strain (%)					
Dapa (+)	–13.70 (–14.63 to –9.98)	–14.15 (–15.35 to –10.95)	–14.50 (–15.70 to –11.1)	<0.001	0.000–0.000
Dapa (−)	–13.70 (–14.90 to –11.3)	–12.90 (–14.75 to –10.75)	–13.2 (–14.65 to –10.15)	0.021	0.017–0.025
Left atrial volume index (mL/m^2^)					
Dapa (+)	42.06 (38.8–49.99)	40.22 (37.36–48.12)	39.51 (35.55–46.57)	<0.001	0.000–0.000
Dapa (−)	41.35 (38.36–46.03)	41.98 (38.26–47.34)	42.54 (38.15–49.25)	0.114	0.106–0.122
Left ventricular mass index (g/m^2^)					
Dapa (+)	94.60 (89.46–105.82)	96.66 (89.62–106.17)	97.12 (89.83–107.23)	0.003	0.001–0.004
Dapa (−)	90.47 (86.37–106.59)	91.68 (87.34–107.64)	96.41 (86.70–106.65)	0.001	0.000–0.000

**Table 4:** Results of the stepwise regression analysis with the change in left ventricular ejection fraction during follow-up as the dependent variable (R^2^ of the model = 0.328, *p* < 0.001).

Independent Variable	Odds Ratio (OR)	95% Confidence Interval of OR	*p*
Treatment with dapagliflozin	57.05	10.65–305.51	<0.001
Age	0.87	0.79–0.95	0.002
Baseline glycated hemoglobin	0.41	0.20–0.87	0.020
Baseline urea	0.98	0.944–1.13	0.199

## Data Availability

The data presented in this study are available upon reasonable request from the corresponding author.

## References

[1] Triposkiadis F., Xanthopoulos A., Bargiota A., Kitai T., Katsiki N., Farmakis D., Skoularigis J., Starling R.C., Iliodromitis E. (2021). Diabetes Mellitus and Heart Failure. J. Clin. Med..

[2] Park J.J. (2021). Epidemiology, Pathophysiology, Diagnosis and Treatment of Heart Failure in Diabetes. Diabetes Metab. J..

[3] Maack C., Lehrke M., Backs J., Heinzel F.R., Hulot J.S., Marx N., Paulus W.J., Rossignol P., Taegtmeyer H., Bauersachs J. (2018). Heart failure and diabetes: Metabolic alterations and therapeutic interventions: A state-of-the-art review from the Translational Research Committee of the Heart Failure Association-European Society of Cardiology. Eur. Heart J..

[4] Braunwald E. (2019). Diabetes, heart failure, and renal dysfunction: The vicious circles. Prog. Cardiovasc. Dis..

[5] Yu Y.W., Zhao X.M., Wang Y.H., Zhou Q., Huang Y., Zhai M., Zhang J. (2021). Effect of sodium-glucose cotransporter 2 inhibitors on cardiac structure and function in type 2 diabetes mellitus patients with or without chronic heart failure: A meta-analysis. Cardiovasc. Diabetol..

[6] Zelniker T.A., Braunwald E. (2020). Mechanisms of Cardiorenal Effects of Sodium-Glucose Cotransporter 2 Inhibitors: JACC State-of-the-Art Review. J. Am. Coll. Cardiol..

[7] Lopaschuk G.D., Verma S. (2020). Mechanisms of Cardiovascular Benefits of Sodium Glucose Co-Transporter 2 (SGLT2) Inhibitors: A State-of-the-Art Review. JACC Basic. Transl. Sci..

[8] Zhang N., Wang Y., Tse G., Korantzopoulos P., Letsas K.P., Zhang Q., Li G., Lip G.Y.H., Liu T. (2022). Effect of sodium-glucose cotransporter-2 inhibitors on cardiac remodelling: A systematic review and meta-analysis. Eur. J. Prev. Cardiol..

[9] Jensen J., Omar M., Kistorp C., Tuxen C., Poulsen M.K., Faber J., Kober L., Gustafsson F., Moller J.E., Schou M. (2022). Effect of Empagliflozin on Multiple Biomarkers in Heart Failure: Insights From the Empire Heart Failure Trial. Circ. Heart Fail..

[10] Lee M.M.Y., Brooksbank K.J.M., Wetherall K., Mangion K., Roditi G., Campbell R.T., Berry C., Chong V., Coyle L., Docherty K.F. (2021). Effect of Empagliflozin on Left Ventricular Volumes in Patients with Type 2 Diabetes, or Prediabetes, and Heart Failure with Reduced Ejection Fraction (SUGAR-DM-HF). Circulation.

[11] Santos-Gallego C.G., Requena-Ibanez J.A., San Antonio R., Ishikawa K., Watanabe S., Picatoste B., Flores E., Garcia-Ropero A., Sanz J., Hajjar R.J. (2019). Empagliflozin Ameliorates Adverse Left Ventricular Remodeling in Nondiabetic Heart Failure by Enhancing Myocardial Energetics. J. Am. Coll. Cardiol..

[12] Omar M., Jensen J., Ali M., Frederiksen P.H., Kistorp C., Videbaek L., Poulsen M.K., Tuxen C.D., Moller S., Gustafsson F. (2021). Associations of Empagliflozin with Left Ventricular Volumes, Mass, and Function in Patients with Heart Failure and Reduced Ejection Fraction: A Substudy of the Empire HF Randomized Clinical Trial. JAMA Cardiol..

[13] Shim C.Y., Seo J., Cho I., Lee C.J., Cho I.J., Lhagvasuren P., Kang S.M., Ha J.W., Han G., Jang Y. (2021). Randomized, Controlled Trial to Evaluate the Effect of Dapagliflozin on Left Ventricular Diastolic Function in Patients with Type 2 Diabetes Mellitus: The IDDIA Trial. Circulation.

[14] Singh J.S.S., Mordi I.R., Vickneson K., Fathi A., Donnan P.T., Mohan M., Choy A.M.J., Gandy S., George J., Khan F. (2020). Dapagliflozin Versus Placebo on Left Ventricular Remodeling in Patients with Diabetes and Heart Failure: The REFORM Trial. Diabetes Care.

[15] Soga F., Tanaka H., Tatsumi K., Mochizuki Y., Sano H., Toki H., Matsumoto K., Shite J., Takaoka H., Doi T. (2018). Impact of dapagliflozin on left ventricular diastolic function of patients with type 2 diabetic mellitus with chronic heart failure. Cardiovasc. Diabetol..

[16] Pascual-Figal D.A., Zamorano J.L., Domingo M., Morillas H., Nunez J., Cobo Marcos M., Riquelme-Perez A., Teis A., Santas E., Caro-Martinez C. (2023). Impact of dapagliflozin on cardiac remodelling in patients with chronic heart failure: The DAPA-MODA study. Eur. J. Heart Fail..

[17] Lang R.M., Badano L.P., Mor-Avi V., Afilalo J., Armstrong A., Ernande L., Flachskampf F.A., Foster E., Goldstein S.A., Kuznetsova T. (2015). Recommendations for cardiac chamber quantification by echocardiography in adults: An update from the American Society of Echocardiography and the European Association of Cardiovascular Imaging. J. Am. Soc. Echocardiogr..

[18] Devereux R.B., Alonso D.R., Lutas E.M., Gottlieb G.J., Campo E., Sachs I., Reichek N. (1986). Echocardiographic assessment of left ventricular hypertrophy: Comparison to necropsy findings. Am. J. Cardiol..

[19] Aimo A., Gaggin H.K., Barison A., Emdin M., Januzzi J.L. (2019). Imaging, Biomarker, and Clinical Predictors of Cardiac Remodeling in Heart Failure with Reduced Ejection Fraction. JACC Heart Fail..

[20] Yamanaka S., Sakata Y., Nochioka K., Miura M., Kasahara S., Sato M., Aoyanagi H., Fujihashi T., Hayashi H., Shiroto T. (2020). Prognostic impacts of dynamic cardiac structural changes in heart failure patients with preserved left ventricular ejection fraction. Eur. J. Heart Fail..

[21] Moady G., Ben Gal T., Atar S. (2023). Sodium-Glucose Co-Transporter 2 Inhibitors in Heart Failure-Current Evidence in Special Populations. Life.

[22] Dyck J.R.B., Sossalla S., Hamdani N., Coronel R., Weber N.C., Light P.E., Zuurbier C.J. (2022). Cardiac mechanisms of the beneficial effects of SGLT2 inhibitors in heart failure: Evidence for potential off-target effects. J. Mol. Cell Cardiol..

[23] Buchan T.A., Ching C., Foroutan F., Malik A., Daza J.F., Hing N.N.F., Siemieniuk R., Evaniew N., Orchanian-Cheff A., Ross H.J. (2022). Prognostic value of natriuretic peptides in heart failure: Systematic review and meta-analysis. Heart Fail. Rev..

[24] Nagarajan V., Hernandez A.V., Tang W.H. (2012). Prognostic value of cardiac troponin in chronic stable heart failure: A systematic review. Heart.

[25] Berg D.D., Docherty K.F., Sattar N., Jarolim P., Welsh P., Jhund P.S., Anand I.S., Chopra V., de Boer R.A., Kosiborod M.N. (2022). Serial Assessment of High-Sensitivity Cardiac Troponin and the Effect of Dapagliflozin in Patients with Heart Failure with Reduced Ejection Fraction: An Analysis of the DAPA-HF Trial. Circulation.

[26] Xue L., Yuan X., Zhang S., Zhao X. (2021). Investigating the Effects of Dapagliflozin on Cardiac Function, Inflammatory Response, and Cardiovascular Outcome in Patients with STEMI Complicated with T2DM after PCI. Evid. Based Complement. Altern. Med..

[27] Kayano H., Koba S., Hirano T., Matsui T., Fukuoka H., Tsuijita H., Tsukamoto S., Hayashi T., Toshida T., Watanabe N. (2020). Dapagliflozin Influences Ventricular Hemodynamics and Exercise-Induced Pulmonary Hypertension in Type 2 Diabetes Patients—A Randomized Controlled Trial. Circ. J..

[28] Theofilis P., Antonopoulos A.S., Katsimichas T., Oikonomou E., Siasos G., Aggeli C., Tsioufis K., Tousoulis D. (2022). The impact of SGLT2 inhibition on imaging markers of cardiac function: A systematic review and meta-analysis. Pharmacol. Res..

[29] Jankauskas S.S., Kansakar U., Varzideh F., Wilson S., Mone P., Lombardi A., Gambardella J., Santulli G. (2021). Heart failure in diabetes. Metabolism.

[30] Wee C.F., Teo Y.H., Teo Y.N., Syn N.L., See R.M., Leong S., Yip A.S.Y., Ong Z.X., Lee C.H., Chan M.Y. (2022). Effects of Sodium/Glucose Cotransporter 2 (SGLT2) Inhibitors on Cardiac Imaging Parameters: A Systematic Review and Meta-analysis of Randomized Controlled Trials. J. Cardiovasc. Imaging.

[31] Brown A.J.M., Gandy S., McCrimmon R., Houston J.G., Struthers A.D., Lang C.C. (2020). A randomized controlled trial of dapagliflozin on left ventricular hypertrophy in people with type two diabetes: The DAPA-LVH trial. Eur. Heart J..

[32] Janwanishstaporn S., Cho J.Y., Feng S., Brann A., Seo J.S., Narezkina A., Greenberg B. (2022). Prognostic Value of Global Longitudinal Strain in Patients with Heart Failure with Improved Ejection Fraction. JACC Heart Fail..

[33] Shah A.M., Claggett B., Sweitzer N.K., Shah S.J., Anand I.S., Liu L., Pitt B., Pfeffer M.A., Solomon S.D. (2015). Prognostic Importance of Impaired Systolic Function in Heart Failure with Preserved Ejection Fraction and the Impact of Spironolactone. Circulation.

[34] Chen J.S., Pei Y., Li C.E., Li N.Y., Guo T., Yu J. (2020). Prognostic value of heart failure echocardiography index in HF patients with preserved, mid-ranged and reduced ejection fraction. BMC Cardiovasc. Disord..

[35] Jariwala P., Jadhav K., Punjani A., Boorugu H., Mari A.R. (2021). ADDition of DAPAgliflozin, Sodium-Glucose Cotransporter-2 Inhibitor to Angiotensin Receptor Blocker-Neprilysin Inhibitors Non-Responders in Patient with Refractory Heart Failure with Reduced Ejection Fraction (ADD DAPA trial). Indian. Heart J..

[36] Oldgren J., Laurila S., Akerblom A., Latva-Rasku A., Rebelos E., Isackson H., Saarenhovi M., Eriksson O., Heurling K., Johansson E. (2021). Effects of 6 weeks of treatment with dapagliflozin, a sodium-glucose co-transporter-2 inhibitor, on myocardial function and metabolism in patients with type 2 diabetes: A randomized, placebo-controlled, exploratory study. Diabetes Obes. Metab..

[37] Eickhoff M.K., Olsen F.J., Frimodt-Moller M., Diaz L.J., Faber J., Jensen M.T., Rossing P., Persson F. (2020). Effect of dapagliflozin on cardiac function in people with type 2 diabetes and albuminuria—A double blind randomized placebo-controlled crossover trial. J. Diabetes Complicat..

[38] Brown A., Gandy S., Mordi I.R., McCrimmon R., Ramkumar P.G., Houston J.G., Struthers A.D., Lang C.C. (2021). Dapagliflozin Improves Left Ventricular Myocardial Longitudinal Function in Patients with Type 2 Diabetes. JACC Cardiovasc. Imaging.

[39] Tanaka H., Soga F., Tatsumi K., Mochizuki Y., Sano H., Toki H., Matsumoto K., Shite J., Takaoka H., Doi T. (2020). Positive effect of dapagliflozin on left ventricular longitudinal function for type 2 diabetic mellitus patients with chronic heart failure. Cardiovasc. Diabetol..

[40] Bhatt A.S., Ambrosy A.P., Velazquez E.J. (2017). Adverse Remodeling and Reverse Remodeling After Myocardial Infarction. Curr. Cardiol. Rep..

[41] Jaiswal A., Nguyen V.Q., Carry B.J., le Jemtel T.H. (2016). Pharmacologic and Endovascular Reversal of Left Ventricular Remodeling. J. Card. Fail..

[42] Porter K.E., Turner N.A. (2009). Cardiac fibroblasts: At the heart of myocardial remodeling. Pharmacol. Ther..

[43] Schirone L., Forte M., Palmerio S., Yee D., Nocella C., Angelini F., Pagano F., Schiavon S., Bordin A., Carrizzo A. (2017). A Review of the Molecular Mechanisms Underlying the Development and Progression of Cardiac Remodeling. Oxid. Med. Cell Longev..

[44] Frantz S., Hundertmark M.J., Schulz-Menger J., Bengel F.M., Bauersachs J. (2022). Left ventricular remodelling post-myocardial infarction: Pathophysiology, imaging, and novel therapies. Eur. Heart J..

[45] Salah H.M., Verma S., Santos-Gallego C.G., Bhatt A.S., Vaduganathan M., Khan M.S., Lopes R.D., Al’Aref S.J., McGuire D.K., Fudim M. (2022). Sodium-Glucose Cotransporter 2 Inhibitors and Cardiac Remodeling. J. Cardiovasc. Transl. Res..

[46] Marketou M., Kontaraki J., Maragkoudakis S., Danelatos C., Papadaki S., Zervakis S., Plevritaki A., Vardas P., Parthenakis F., Kochiadakis G. (2022). Effects of Sodium-Glucose Cotransporter-2 Inhibitors on Cardiac Structural and Electrical Remodeling: From Myocardial Cytology to Cardiodiabetology. Curr. Vasc. Pharmacol..

[47] Marfella R., Scisciola L., D’Onofrio N., Maiello C., Trotta M.C., Sardu C., Panarese I., Ferraraccio F., Capuano A., Barbieri M. (2022). Sodium-glucose cotransporter-2 (SGLT2) expression in diabetic and non-diabetic failing human cardiomyocytes. Pharmacol. Res..

[48] Seferovic P.M., Fragasso G., Petrie M., Mullens W., Ferrari R., Thum T., Bauersachs J., Anker S.D., Ray R., Cavusoglu Y. (2020). Sodium-glucose co-transporter 2 inhibitors in heart failure: Beyond glycaemic control. A position paper of the Heart Failure Association of the European Society of Cardiology. Eur. J. Heart Fail..

[49] Correale M., Lamacchia O., Ciccarelli M., Dattilo G., Tricarico L., Brunetti N.D. (2023). Vascular and metabolic effects of SGLT2i and GLP-1 in heart failure patients. Heart Fail. Rev..

[50] Arow M., Waldman M., Yadin D., Nudelman V., Shainberg A., Abraham N.G., Freimark D., Kornowski R., Aravot D., Hochhauser E. (2020). Sodium-glucose cotransporter 2 inhibitor Dapagliflozin attenuates diabetic cardiomyopathy. Cardiovasc. Diabetol..

[51] Fonseca-Correa J.I., Correa-Rotter R. (2021). Sodium-Glucose Cotransporter 2 Inhibitors Mechanisms of Action: A Review. Front. Med..

[52] Chen S., Coronel R., Hollmann M.W., Weber N.C., Zuurbier C.J. (2022). Direct cardiac effects of SGLT2 inhibitors. Cardiovasc. Diabetol..

[53] Joshi S.S., Singh T., Newby D.E., Singh J. (2021). Sodium-glucose co-transporter 2 inhibitor therapy: Mechanisms of action in heart failure. Heart.

